# Supplementary material for: Induction of memory-like dendritic cell responses in vivo
Source: Nat Commun. 2019 Jul 4;10:2955. doi: 10.1038/s41467-019-10486-5 (PMC6609631; doi:10.1038/s41467-019-10486-5)
Supplement: Supplementary file 1 — Supplementary Information [file 41467_2019_10486_MOESM1_ESM.pdf]

Supplementary Information

Induction of Memory-like Dendritic Cell Responses in vivo

Floyd L. Wormley, Jr. et al.

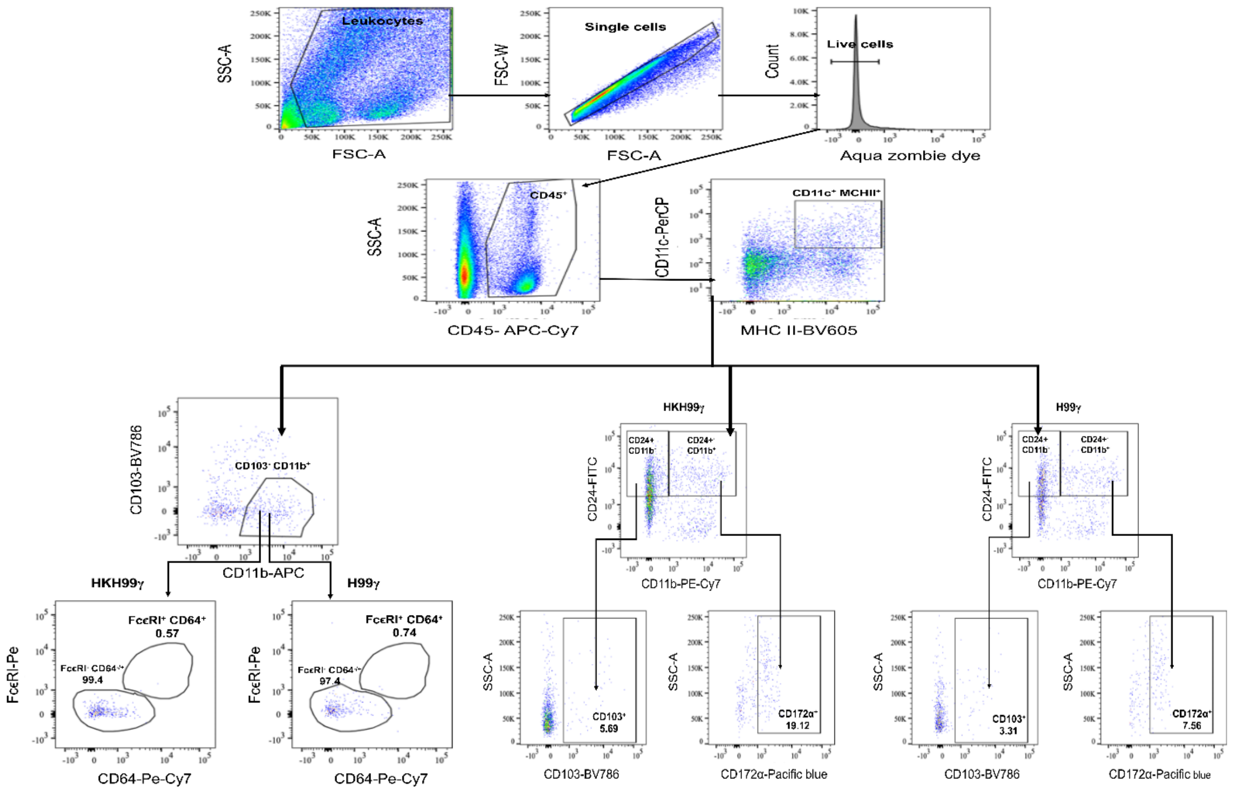

Supplementary Figure 1. Gating strategy for lung conventional dendritic cells. BALB/c mice were immunized via intranasal inhalation with 10^4 CFU of *C. neoformans* strain H99γ or HKH99γ in 50 µl of sterile PBS. Seventy days later, mice were challenged with 10^4 CFU of *C. neoformans* strain H99 via intranasal inhalation. Pulmonary leukocytes were isolated from lung tissues by enzymatic digestion on day 1 post-challenge. Flow cytometry was used to determine the percentage of $CD103^+$ cDCs ($CD11c^+/CD24^+/CD11b^-/CD103^+$), $CD11b^+$cDCs ($CD11c^+/CD24^+/CD11b^+/CD172\alpha^+$) and $CD11b^+$ monocyte-derived DCs ($CD11c^+/CD11b^+/CD24^-/CD64^+$/ $Fc\varepsilon R1\alpha^+/Ly6C^+$/I-A/I-$E^+$) in the lung. Results shown are representative of four individual experiments using 5 mice per group.

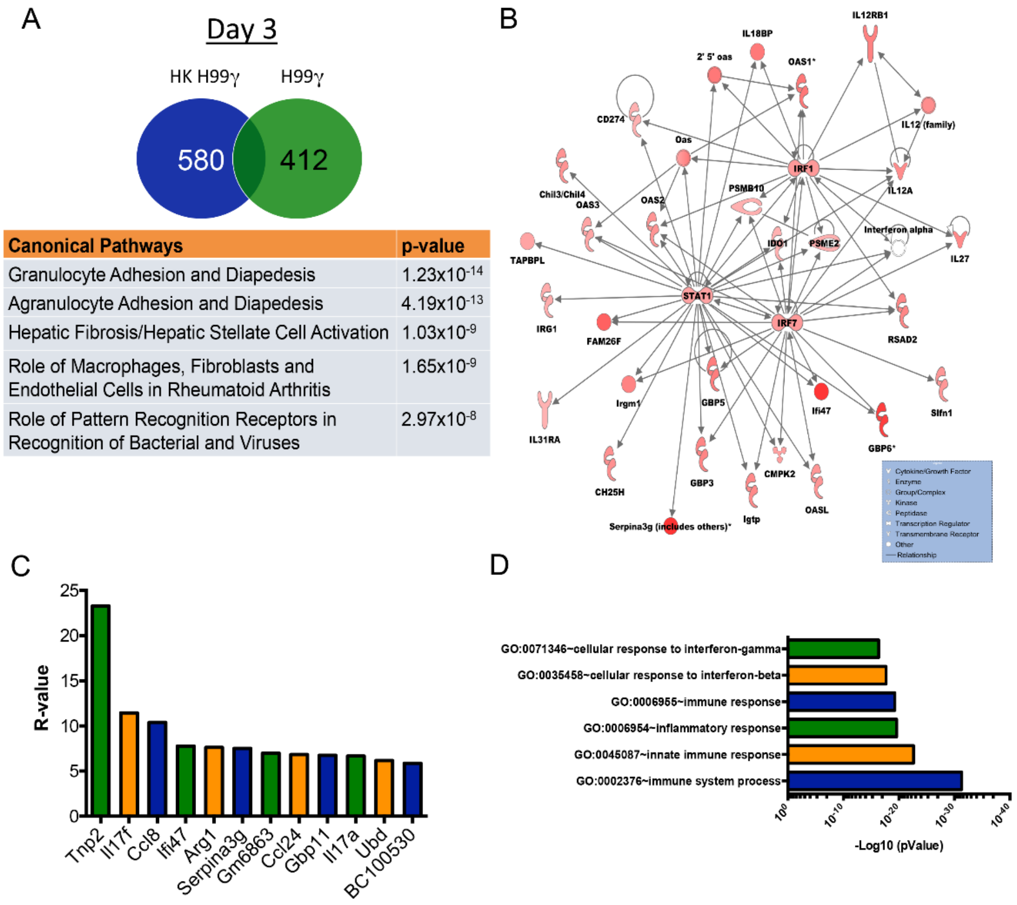

Supplementary Figure 2. DCs from protectively immunized mice exhibit an interferon gamma response network gene expression profile. BALB/c mice received an intranasal immunization with 10^4 CFU of *C. neoformans* strain H99γ or HKH99γ in 50 µl of sterile PBS. Seventy days later, mice were subsequently infected with 10^4 CFU of *C. neoformans* strain H99. Pulmonary leukocytes were isolated from lung tissues by enzymatic digestion on day 3 post-inoculation. Leukocyte populations were depleted of $CD3^+$ cells by positive selection using α-CD3e labeled magnetic beads and macrophages by positive selection using biotinylated α-F4/80 and α-biotin magnetic beads. DCs were isolated by positive selection using $CD11c^+$ magnetic beads. Total mRNA from isolated DC populations was extracted and RNA-seq was performed. **A.** Differently expressed genes and top canonical pathways and networks predicted by the Ingenuity Pathway Analysis software protectively immunized mice compared to non-protectively immunized mice as ranked by p-value. **B**. Top network up-regulated in day 3 post-challenge pulmonary DCs from protectively immunized mice compared to non-protectively immunized mice. The red color in the network figure corresponds to the log2 of expression fold change and represents increased gene expression in DCs from protectively compared to non-protectively immunized mice. **C**. Fold change values for top 12 up-regulated genes from H99γ immunized DCs day 3 post-challenge. **D.** Top 6 GO terms from H99γ immunized DCs day 3 post-challenge. Data were generated from a merged data set from 3 independent experiments with 20-25 mice per group.

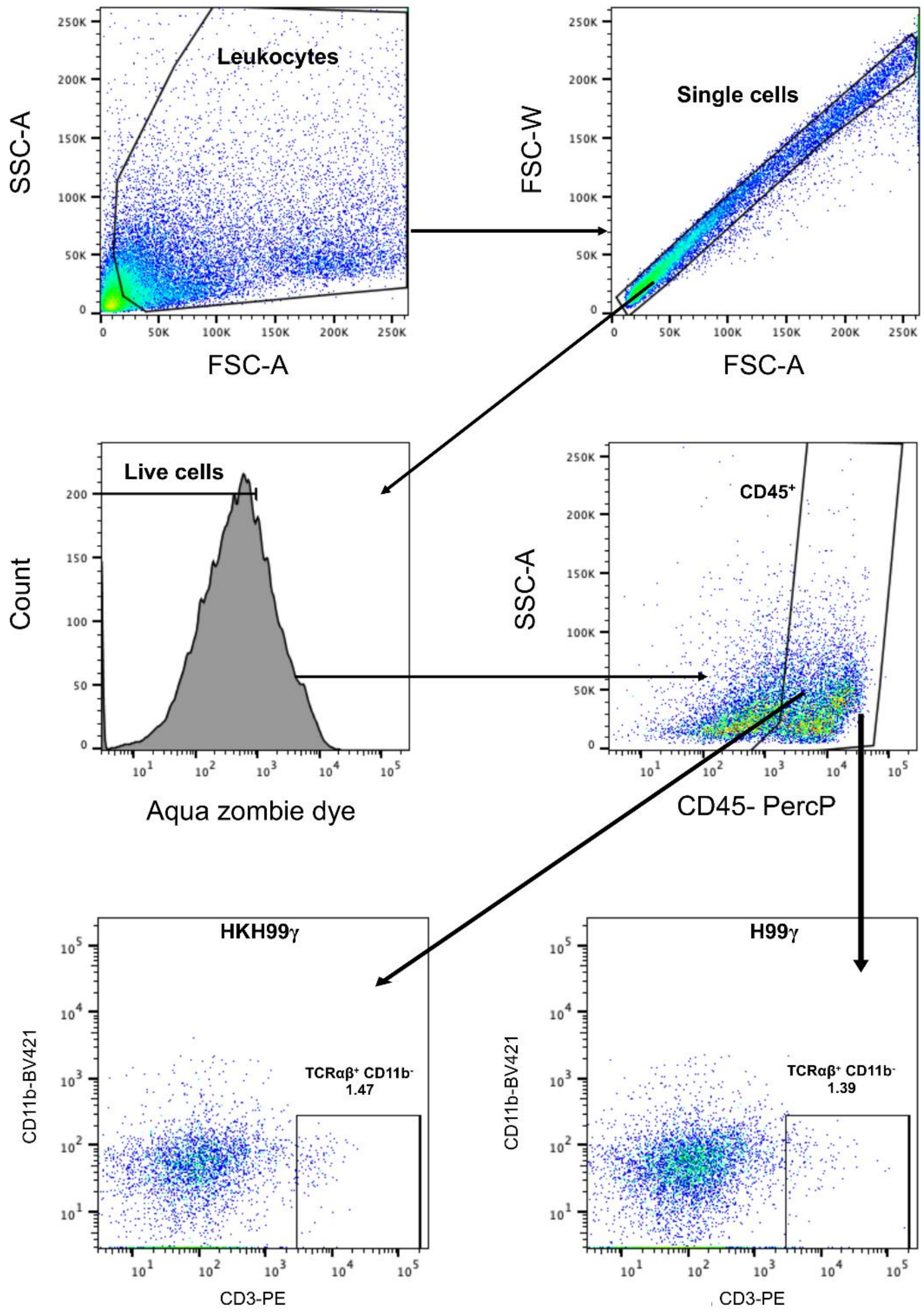

Supplementary Figure 3. Presence of TCRα/β⁺ cells in dendritic cell enriched population. BALB/c mice were intranasally immunized with 10^4 CFU of *C. neoformans* strain H99γ or HKH99γ in 50 µl of sterile PBS. After 70 days, DCs were isolated from the spleens of HKH99γ immunized or H99γ immunized mice after depletion of $CD3^+$ and $F4/80^+$ cells. Flow cytometry was used to determine percentage of $TCR\alpha/\beta^+/CD11b^-$ cells in the enriched DC population derived from the spleens of immunized, unchallenged mice. Results shown are representative of three individual experiments using 3-4 mice per group.

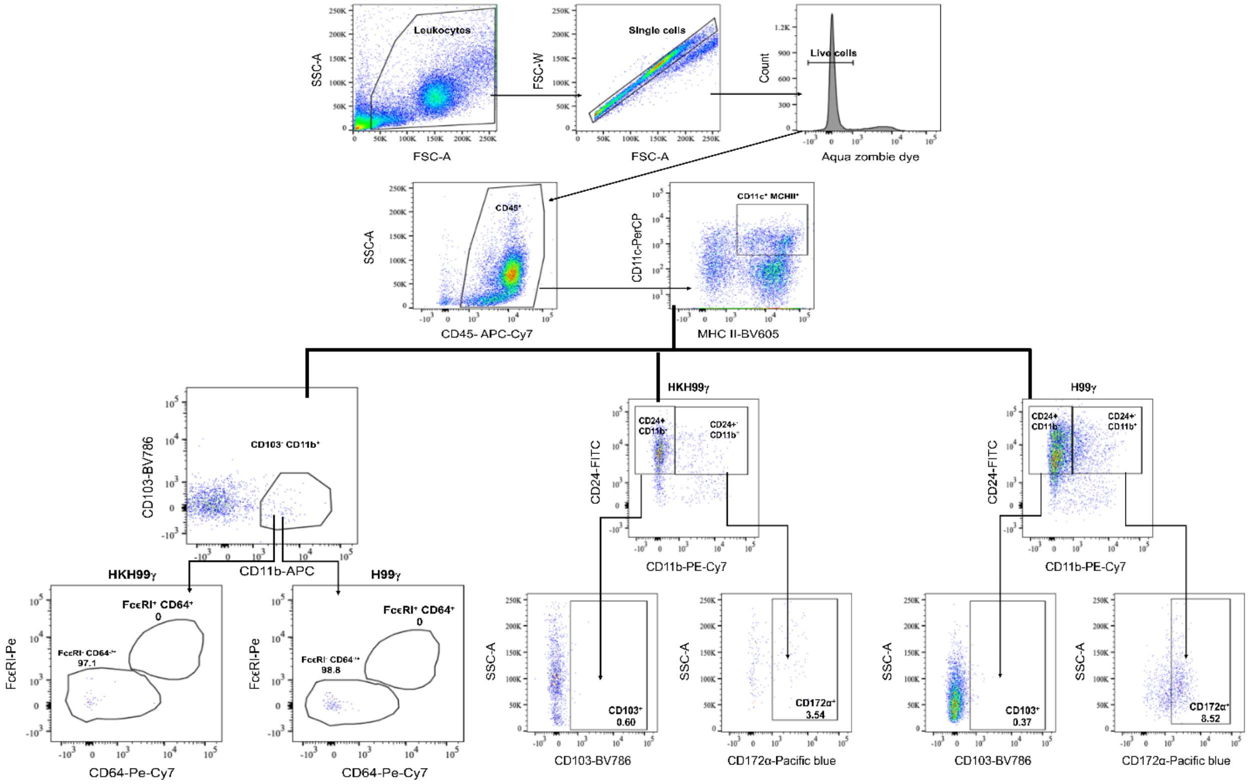

Supplementary Figure 4. Phenotype of splenic dendritic cell enriched population. BALB/c mice were intranasally immunized with 10^4 CFU of *C. neoformans* strain H99γ or HKH99γ in 50 µl of sterile PBS. After 70 days, DCs were isolated from the spleens of HKH99γ immunized or H99γ immunized mice after depletion of $CD3^+$ and $F4/80^+$ cells. Flow cytometry was used to determine percentage of $CD103^+$ cDCs ($CD11c^+/CD24^+/CD11b^-/CD103^+$), $CD11b^+$cDCs ($CD11c^+/CD24^+/CD11b^+/CD172\alpha^+$) and $CD11b^+$ monocyte-derived DCs ($CD11c^+/CD11b^+/CD24^-/CD64^+/$ $Fc\varepsilon R1\alpha^+/Ly6C^+/I\text{-}A/I\text{-}E^+$) in the enriched DC population derived from the spleens of immunized, unchallenged mice. Results shown are representative of three individual experiments using 3-4 mice per group.

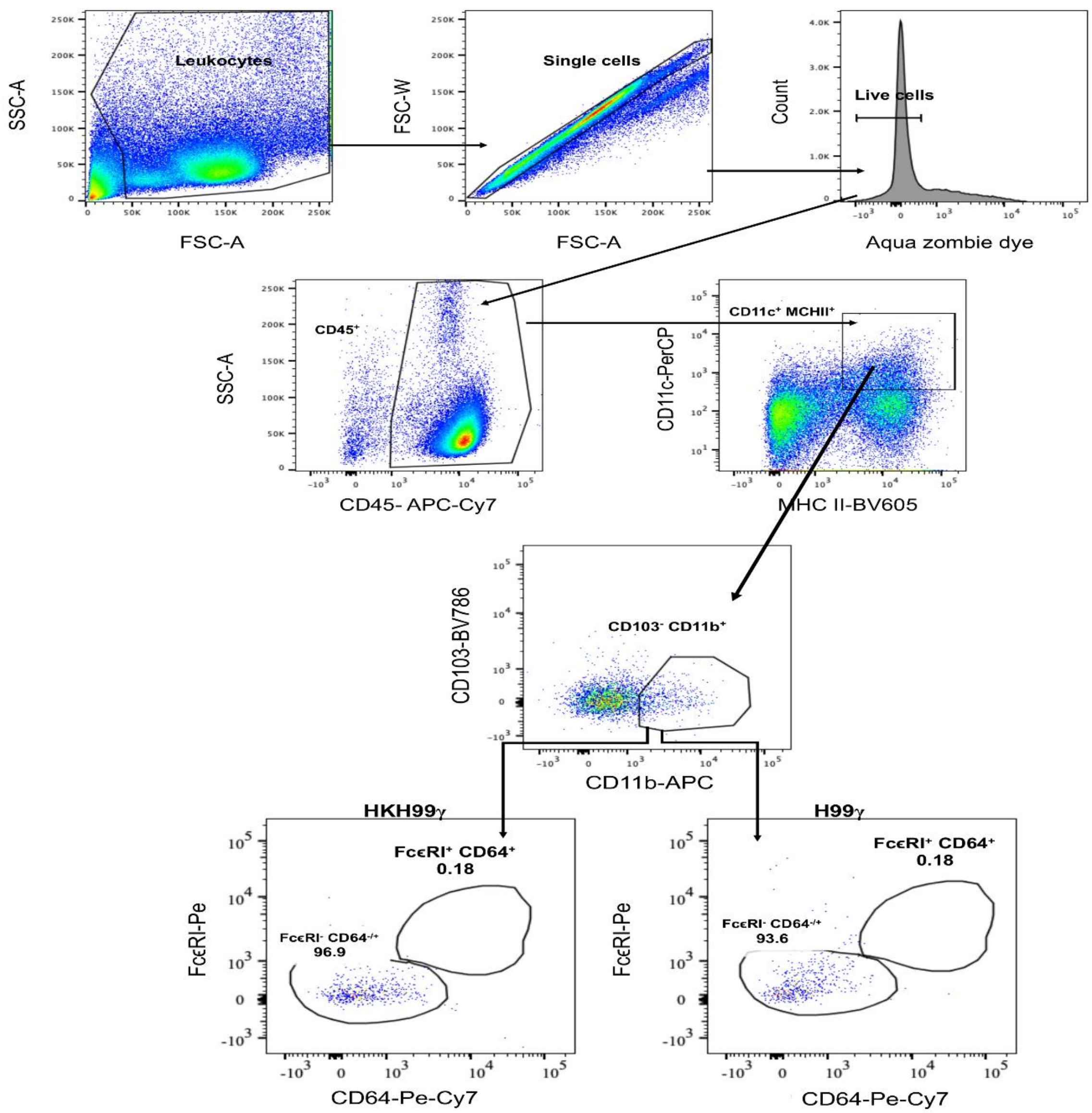

Supplementary Figure 5. Phenotype of splenic dendritic cell population. BALB/c mice were intranasally immunized with 10^4 CFU of *C. neoformans* strain H99γ or HKH99γ in 50 µl of sterile PBS. After 70 days, DCs were isolated from the spleens of HKH99γ immunized or H99γ immunized mice. Flow cytometry was used to determine percentage of $CD11b^+$ monocyte-derived DCs ($CD11c^+$/$CD11b^+$/$CD103^-$/$CD64^+$/ $Fc\varepsilon R1\alpha^+$/$Ly6C^+$/I-A/$I\text{-}E^+$) in cell population derived from the spleens of immunized, unchallenged mice. Results shown are representative of three individual experiments using 3-4 mice per group.

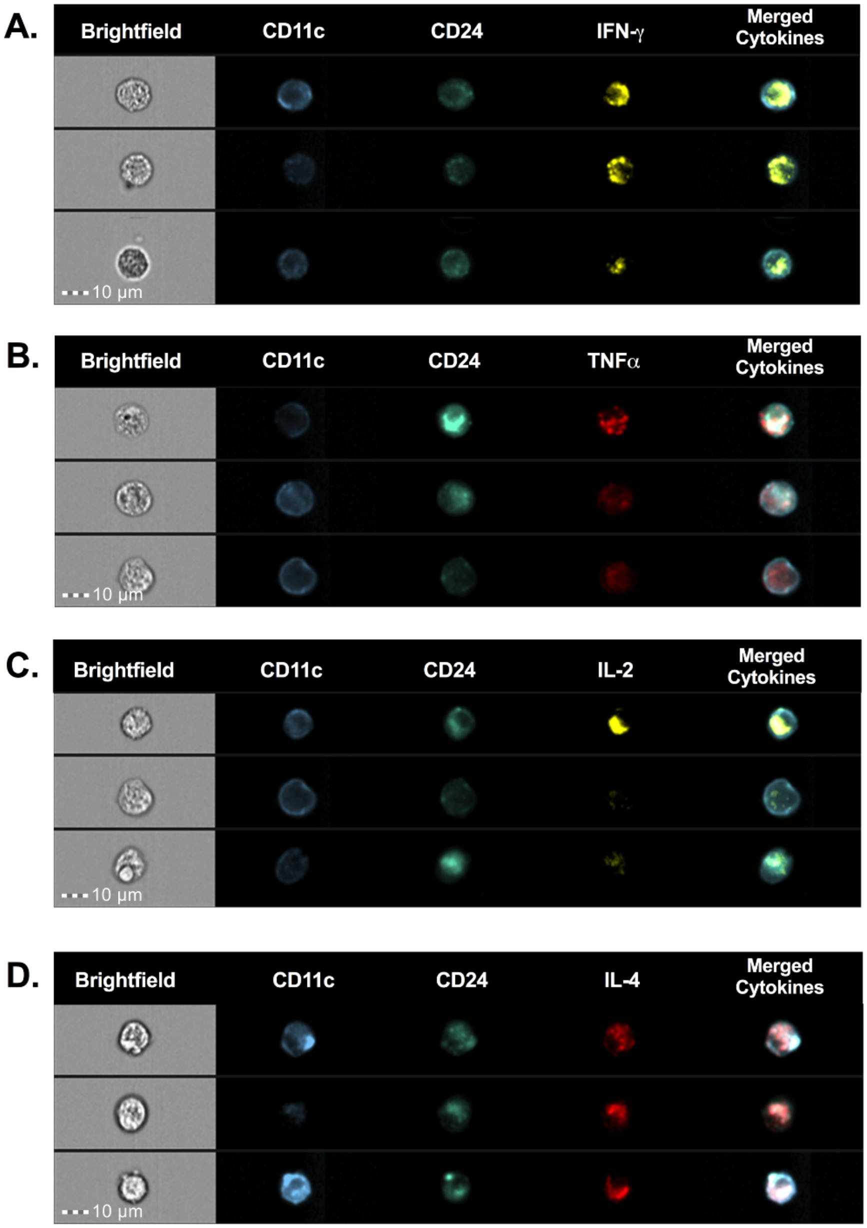

Supplementary Figure 6. Flow cytometry analysis of intracellular cytokine production. BALB/c mice were immunized with *C. neoformans* strain H99γ and rested for 70 days. Dendritic cells were isolated from the spleens and cultured *ex vivo* with *C. neoformans cna1*Δ. Production of cytokines **A-D.** IFN-γ, TNF-α, IL-2, and IL-4 were verified by intracellular flow cytometry after 6 hours of culture. Data shown is representative of 3 individual experiments with 10 mice per group.

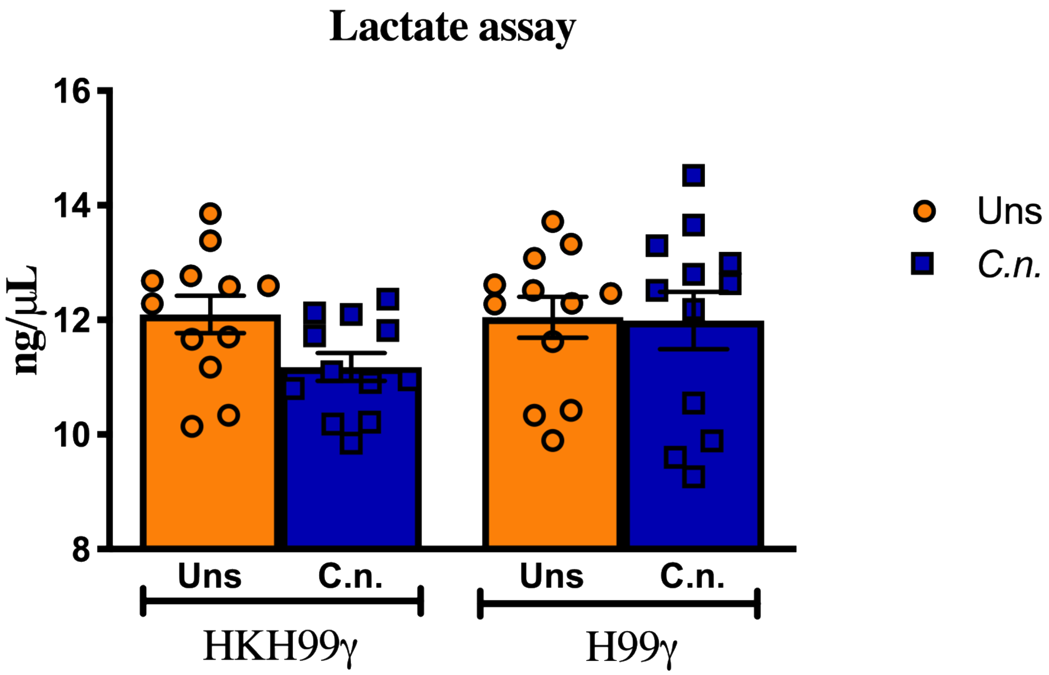

Supplementary Figure 7. Lactate release by dendritic cells. Dendritic cells (DCs) were isolated from the lungs of BALB/c mice intranasally immunized with 10^4 CFU of *C. neoformans* strain H99γ or HKH99γ in 50 µl of sterile PBS. After 70 days, DCs were isolated from the lungs and cultured in RPMI complete media alone (Uns) or media + *C. neoformans* calcineurin mutant (C.n.) for 24 hours at 37°C in 5% CO_2. Subsequently, the supernatant was collected lactate dehydrogenase removed and L-lactate concentration determined. No significant difference in L-lactate release was observed between groups. Error bars are +/- standard error of the mean (SEM). one-way ANOVA
