# Supplementary material for: Induction of memory-like dendritic cell responses in vivo
Source: Nat Commun. 2019 Jul 4;10:2955. doi: 10.1038/s41467-019-10486-5 (PMC6609631; doi:10.1038/s41467-019-10486-5)
Supplement: Supplementary file 3 — Reporting Summary [file 41467_2019_10486_MOESM3_ESM.pdf]

nature research

Corresponding author(s): *Double-blind peer review submissions: write DBPR and your manuscript number here instead of author names.*

Last updated by author(s): *YYYY-MM-DD*

Reporting Summary

Nature Research wishes to improve the reproducibility of the work that we publish. This form provides structure for consistency and transparency in reporting. For further information on Nature Research policies, see Authors & Referees and the Editorial Policy Checklist.

Statistics

For all statistical analyses, confirm that the following items are present in the figure legend, table legend, main text, or Methods section.

n/a	Confirmed	
☐	☒	The exact sample size (n) for each experimental group/condition, given as a discrete number and unit of measurement
☐	☒	A statement on whether measurements were taken from distinct samples or whether the same sample was measured repeatedly
☐	☒	The statistical test(s) used AND whether they are one- or two-sided<br>*Only common tests should be described solely by name; describe more complex techniques in the Methods section.*
☒	☐	A description of all covariates tested
☐	☒	A description of any assumptions or corrections, such as tests of normality and adjustment for multiple comparisons
☐	☒	A full description of the statistical parameters including central tendency (e.g. means) or other basic estimates (e.g. regression coefficient) AND variation (e.g. standard deviation) or associated estimates of uncertainty (e.g. confidence intervals)
☐	☒	For null hypothesis testing, the test statistic (e.g. F, t, r) with confidence intervals, effect sizes, degrees of freedom and P value noted<br>*Give P values as exact values whenever suitable.*
☒	☐	For Bayesian analysis, information on the choice of priors and Markov chain Monte Carlo settings
☒	☐	For hierarchical and complex designs, identification of the appropriate level for tests and full reporting of outcomes
☒	☐	Estimates of effect sizes (e.g. Cohen's d, Pearson's r), indicating how they were calculated

Our web collection on statistics for biologists contains articles on many of the points above.

Software and code

Policy information about availability of computer code

Data collection	Flow Cytometry: BD FACSDiva software<br>Intracellular Flow Cytometry: ImageStreamX IDEAS® 6.2 software (Millipore, Burlington, MA)<br>No custom code/software was used
Data analysis	Statistics: GraphPad Prism version 5.00 for Windows (GraphPad Prism Software, San Diego California)<br>Gene ontology analysis: DAVID functional analysis tool<br>Functional pathway and network analyse: Ingenuity Pathway Analysis (IPA) (Qiagen, Germantown, MD)<br>No custom code/software was used

For manuscripts utilizing custom algorithms or software that are central to the research but not yet described in published literature, software must be made available to editors/reviewers. We strongly encourage code deposition in a community repository (e.g. GitHub). See the Nature Research guidelines for submitting code & software for further information.

Data

Policy information about availability of data

All manuscripts must include a data availability statement. This statement should provide the following information, where applicable:

- Accession codes, unique identifiers, or web links for publicly available datasets
- A list of figures that have associated raw data
- A description of any restrictions on data availability

The RNA-Seq results presented in this manuscript have been submitted to the Sequence Read Archive under accession number SRP120338SUB310649 [https://www.ncbi.nlm.nih.gov/sra/?term=SRP120338]. The authors declare that all data supporting the findings of this study are available within the article and its supplementary information files or from the corresponding author upon reasonable request.

Field-specific reporting

Please select the one below that is the best fit for your research. If you are not sure, read the appropriate sections before making your selection.

☒ Life sciences ☐ Behavioural & social sciences ☐ Ecological, evolutionary & environmental sciences

For a reference copy of the document with all sections, see nature.com/documents/nr-reporting-summary-flat.pdf

Life sciences study design

All studies must disclose on these points even when the disclosure is negative.

Sample size

Sample size was determined to obtain sufficient quantity of cells/material to perform experiments (i.e., obtain RNA or cells to perform cell culture experiments) and numbers of mice per group also done in consideration of group sizes sufficient to support our conclusions with statistical significance. Power analysis was performed when appropriate based on previous studies to detect a 10% change with alpha of 0.05.

Data exclusions

No data were excluded from analysis

Replication

Independent experiments were successfully replicated at least twice and more than twice in most cases. The exact numbers of biologically independent repetitions are provided in the Methods section and/or Figure Legends of the manuscript. Controls were compared to previous results of similar studies to confirm reproducibility of data when possible. Observations appeared to be reproducible and are reported as observed.

Randomization

Mice were randomly assigned to groups and these groupings were maintained throughout the individual experiments in order to comply with the experimental design (immunized vs. mock-immunized).

Blinding

Investigators were not blinded as to mouse group allocations (not a clinical study) or collections as we needed to remain mindful of immunization groups to comply with study design and to ensure appropriate handling and data acquisition. Nonetheless, more than one scientist performed the reported experiments and analyzed the data independently. Also, data analysis was performed by computer-based methods and no data was excluded from study. Thus, blinding was not necessary to our studies.

Reporting for specific materials, systems and methods

We require information from authors about some types of materials, experimental systems and methods used in many studies. Here, indicate whether each material, system or method listed is relevant to your study. If you are not sure if a list item applies to your research, read the appropriate section before selecting a response.

Materials & experimental systems

n/a	Involved in the study
☐	☒ Antibodies
☐	☐ Eukaryotic cell lines
☐	☐ Palaeontology
☐	☒ Animals and other organisms
☐	☐ Human research participants
☐	☐ Clinical data

Methods

n/a	Involved in the study
☐	☐ ChIP-seq
☐	☒ Flow cytometry
☐	☐ MRI-based neuroimaging

Antibodies

Antibodies used

DC enrichment: biotinylated α-F4/80 (Cat. No 13-4801-85, clone BM8, Affymetrix eBioscience Inc. San Diego, CA)
Flow cytometry: Fc block (1:500 dilution, Cat. No 553142, clone 2.4G2, BD Biosciences)
CD45-BV711 (1:200 dilution, Cat. No 103147, clone 30-F11, Biolegend)
CD11b-APC (1:200 dilution, Cat. No 553312, clone M1/70, BD Biosciences)
CD11c-PerCP (1:5:100 dilution, Cat. No 45-914-80, clone N418, eBioscience)
CD24-FITC (1:250 dilution, Cat. No 561777, clone M1/69, BD Bioscience)
CD64-PeCy7 (1:100 dilution, Cat. No 139313, clone X54-5/7.1, Biolegend)
FcεR1alpha-PE (1:200, Cat. No 12-5898-81, clone MAR-1, eBioscience)
Ly6C-BV421 (1:200 dilution, Cat. No 562727, clone AL-21, BD Horizon)
I-A/I-E-BV605 (1:100 dilution, Cat. No 563413, clone M5/114.15.2, BD Horizon)
CD103-BV786 (1:100 dilution, Cat No, 564322, clone M290, BD Bioscience)
CD172alpha (1:100 dilution, Cat No. 740071, clone P84, BD Bioscience)
TCRalpha/beta-PE (1:200 dilution, Cat. No 553172, clone H57-597, BD Bioscience)
IFN-γ-APC (1:400 dilution; Cat. No 17-7311-81, clone XMG1.2, eBioscience)
IL-2-PE (1:400 dilution; Cat. No 561061, clone JES6-5H4, BD Biosciences)
TNF-α-PE (1:400 dilution; Cat. No 12-7321-81, clone MP6-XT22, eBioscience)
IL-4-APC (1:400 dilution; Cat. No 17-7041-81, clone 11B11, eBioscience)

Validation	No customized antibodies were utilized in this study. Validation data of the antibodies purchased from commercial vendors are available on the vendor's web sites and/or data sheets that accompanied the antibodies. Murine cells known to be positive for target antigens/proteins were used to validate antibodies used for flow cytometry.

Eukaryotic cell lines

Policy information about cell lines

Cell line source(s)	*State the source of each cell line used.*
Authentication	*Describe the authentication procedures for each cell line used OR declare that none of the cell lines used were authenticated.*
Mycoplasma contamination	*Confirm that all cell lines tested negative for mycoplasma contamination OR describe the results of the testing for mycoplasma contamination OR declare that the cell lines were not tested for mycoplasma contamination.*
Commonly misidentified lines (See ICLAC register)	*Name any commonly misidentified cell lines used in the study and provide a rationale for their use.*

Palaeontology

Specimen provenance	*Provide provenance information for specimens and describe permits that were obtained for the work (including the name of the issuing authority, the date of issue, and any identifying information).*
Specimen deposition	*Indicate where the specimens have been deposited to permit free access by other researchers.*
Dating methods	*If new dates are provided, describe how they were obtained (e.g. collection, storage, sample pretreatment and measurement), where they were obtained (i.e. lab name), the calibration program and the protocol for quality assurance OR state that no new dates are provided.*

☐ Tick this box to confirm that the raw and calibrated dates are available in the paper or in Supplementary Information.

Animals and other organisms

Policy information about studies involving animals; ARRIVE guidelines recommended for reporting animal research

Laboratory animals	Female BALB/c (H-2d) mice between 5-6 weeks of age
Wild animals	Study did not involved wild animals
Field-collected samples	Study did not involve samples collected from the field
Ethics oversight	Institutional Animal Care and Use Committee and Institutional Biosafety Committee of of the University of Texas at San Antonio

Note that full information on the approval of the study protocol must also be provided in the manuscript.

Human research participants

Policy information about studies involving human research participants

Population characteristics	*Describe the covariate-relevant population characteristics of the human research participants (e.g. age, gender, genotypic information, past and current diagnosis and treatment categories). If you filled out the behavioural & social sciences study design questions and have nothing to add here, write "See above."*
Recruitment	*Describe how participants were recruited. Outline any potential self-selection bias or other biases that may be present and how these are likely to impact results.*
Ethics oversight	*Identify the organization(s) that approved the study protocol.*

Note that full information on the approval of the study protocol must also be provided in the manuscript.

Clinical data

Policy information about clinical studies

All manuscripts should comply with the ICMJE guidelines for publication of clinical research and a completed CONSORT checklist must be included with all submissions.

Clinical trial registration	*Provide the trial registration number from ClinicalTrials.gov or an equivalent agency.*

Study protocol | Note where the full trial protocol can be accessed OR if not available, explain why.

Data collection | Describe the settings and locales of data collection, noting the time periods of recruitment and data collection.

Outcomes | Describe how you pre-defined primary and secondary outcome measures and how you assessed these measures.

ChIP-seq

Data deposition

☐ Confirm that both raw and final processed data have been deposited in a public database such as GEO.

☐ Confirm that you have deposited or provided access to graph files (e.g. BED files) for the called peaks.

Data access links<br>*May remain private before publication.*	*For "Initial submission" or "Revised version" documents, provide reviewer access links. For your "Final submission" document, provide a link to the deposited data.*
Files in database submission	*Provide a list of all files available in the database submission.*
Genome browser session<br>(e.g. UCSC)	*Provide a link to an anonymized genome browser session for "Initial submission" and "Revised version" documents only, to enable peer review. Write "no longer applicable" for "Final submission" documents.*

Methodology

Replicates	*Describe the experimental replicates, specifying number, type and replicate agreement.*
Sequencing depth	*Describe the sequencing depth for each experiment, providing the total number of reads, uniquely mapped reads, length of reads and whether they were paired- or single-end.*
Antibodies	*Describe the antibodies used for the ChIP-seq experiments; as applicable, provide supplier name, catalog number, clone name, and lot number.*
Peak calling parameters	*Specify the command line program and parameters used for read mapping and peak calling, including the ChIP, control and index files used.*
Data quality	*Describe the methods used to ensure data quality in full detail, including how many peaks are at FDR 5% and above 5-fold enrichment.*
Software	*Describe the software used to collect and analyze the ChIP-seq data. For custom code that has been deposited into a community repository, provide accession details.*

Flow Cytometry

Plots

Confirm that:

☒ The axis labels state the marker and fluorochrome used (e.g. CD4-FITC).

☒ The axis scales are clearly visible. Include numbers along axes only for bottom left plot of group (a 'group' is an analysis of identical markers).

☒ All plots are contour plots with outliers or pseudocolor plots.

☒ A numerical value for number of cells or percentage (with statistics) is provided.

Methodology

Sample preparation	*Describe the sample preparation, detailing the biological source of the cells and any tissue processing steps used.*
Instrument	Cell Analyzer LSRII (BD Biosciences); ImageStreamX ISX-MKII Imaging Flow Cytometer (Luminex Corporation, Seattle, WA)
Software	FlowJo v.10 Software (FlowJo, LLC, Ashland, OR); BD FACSDiva v8.0 (BD Biosciences); IDEAS® v6.2 software (Luminex Corporation)
Cell population abundance	For data analyses, 100,000 events (cells) were evaluated. Dendritic cell purity were assessed by flow cytometry analysis to be > 95% pure and contain < 1.5% TCRalpha/beta+ cells.
Gating strategy	Fluorescence minus one (FMO) controls or cells incubated with either FACS buffer alone or single fluorochrome-conjugated Abs were used to determine positive staining and spillover/compensation calculations, and background fluorescence determined with FlowJo v.10 Software (FlowJo, LLC, Ashland, OR). Raw data was collected with a Cell Analyzer LSRII (BD Biosciences) using BD FACSDiva v8.0 software at the Cell Analysis Core of the UTSA and compensation and data analyses were performed using FlowJo v.10 Software. Cells were first gated for lymphocytes (SSC-A vs FSC-A), and for singlets (FSC-H vs. FSC-A). The singlets gate was

further analyzed for the uptake of live/dead yellow stain to determine live vs. dead cells. From live cells, cells were gated on CD45+ cell expression. For data analyses, 100,000 events (cells) were evaluated from a predominantly leukocyte population identified by back gating from CD45+ stained cells. The absolute number of total dendritic cells was quantified by multiplying the total number of cells observed by hemacytometer counting by the percentage of CD45+ cells determined by flow cytometry.

☒ Tick this box to confirm that a figure exemplifying the gating strategy is provided in the Supplementary Information.

Magnetic resonance imaging

Experimental design

Design type — *Indicate task or resting state; event-related or block design.*

Design specifications — *Specify the number of blocks, trials or experimental units per session and/or subject, and specify the length of each trial or block (if trials are blocked) and interval between trials.*

Behavioral performance measures — *State number and/or type of variables recorded (e.g. correct button press, response time) and what statistics were used to establish that the subjects were performing the task as expected (e.g. mean, range, and/or standard deviation across subjects).*

Acquisition

Imaging type(s) — *Specify: functional, structural, diffusion, perfusion.*

Field strength — *Specify in Tesla*

Sequence & imaging parameters — *Specify the pulse sequence type (gradient echo, spin echo, etc.), imaging type (EPI, spiral, etc.), field of view, matrix size, slice thickness, orientation and TE/TR/flip angle.*

Area of acquisition — *State whether a whole brain scan was used OR define the area of acquisition, describing how the region was determined.*

Diffusion MRI ☐ Used ☐ Not used

Preprocessing

Preprocessing software — *Provide detail on software version and revision number and on specific parameters (model/functions, brain extraction, segmentation, smoothing kernel size, etc.).*

Normalization — *If data were normalized/standardized, describe the approach(es): specify linear or non-linear and define image types used for transformation OR indicate that data were not normalized and explain rationale for lack of normalization.*

Normalization template — *Describe the template used for normalization/transformation, specifying subject space or group standardized space (e.g. original Talairach, MNI305, ICBM152) OR indicate that the data were not normalized.*

Noise and artifact removal — *Describe your procedure(s) for artifact and structured noise removal, specifying motion parameters, tissue signals and physiological signals (heart rate, respiration).*

Volume censoring — *Define your software and/or method and criteria for volume censoring, and state the extent of such censoring.*

Statistical modeling & inference

Model type and settings — *Specify type (mass univariate, multivariate, RSA, predictive, etc.) and describe essential details of the model at the first and second levels (e.g. fixed, random or mixed effects; drift or auto-correlation).*

Effect(s) tested — *Define precise effect in terms of the task or stimulus conditions instead of psychological concepts and indicate whether ANOVA or factorial designs were used.*

Specify type of analysis: ☐ Whole brain ☐ ROI-based ☐ Both

Statistic type for inference (See Eklund et al. 2016) — *Specify voxel-wise or cluster-wise and report all relevant parameters for cluster-wise methods.*

Correction — *Describe the type of correction and how it is obtained for multiple comparisons (e.g. FWE, FDR, permutation or Monte Carlo).*

Models & analysis

n/a	Involved in the study
☐	☐ Functional and/or effective connectivity
☐	☐ Graph analysis
☐	☐ Multivariate modeling or predictive analysis

Functional and/or effective connectivity	*Report the measures of dependence used and the model details (e.g. Pearson correlation, partial correlation, mutual information).*
Graph analysis	*Report the dependent variable and connectivity measure, specifying weighted graph or binarized graph, subject- or group-level, and the global and/or node summaries used (e.g. clustering coefficient, efficiency, etc.).*
Multivariate modeling and predictive analysis	*Specify independent variables, features extraction and dimension reduction, model, training and evaluation metrics.*
